# Evaluation of the treatment results in patients with rhegmatogenous retinal detachment treated by pars plana vitrectomy with air: Preliminary results

**DOI:** 10.1097/MD.0000000000010902

**Published:** 2018-06-01

**Authors:** Katarzyna Michalska-Małecka, Justyna Sierocka-Stępień, Aneta Michalik-Jakubek, Katarzyna Witek, Katarzyna Nowomiejska, Anselm Jünemann, Robert Rejdak

**Affiliations:** a Department of Ophthalmology, School of Medicine in Katowice; b Professor K. Gibinski University Clinical Center, Medical University of Silesia, Katowice; c Department of General Ophthalmology, Medical University in Lublin, Lublin, Poland; d Department of Ophthalmology, University Eye Hospital, Rostock, Germany.

**Keywords:** air, endotamponade, pars plana vitrectomy, rhegmatogenous retinal detachment, silicone oil

## Abstract

**Purpose:**

The aim of this study was to evaluate the effectiveness of treatment and the restoration of the anatomic conditions (retinal reattachment) in rhegmatogenous retinal detachment treated by pars plana vitrectomy (PPV) using air as endotamponade.

**Methods:**

Twelve patients (12 eyes) with rhegmatogenous retinal detachment were included in the study. These were eyes with retinal detachment both with and without macular involvement. During the PPV surgery, the peripheral and central part of the vitreous body was excised to eliminate the formation of vitreoretinal traction after surgery, laser therapy was performed peripherally and in retinal openings, and the eyeball was filled with filtered air. Local conditions were evaluated at 1, 7, 14, 30, 90, and 180 days after the procedure.

**Results:**

The result of the patients’ examination on the 30^th^ day after surgery showed that 100% of cases achieved retinal reattachment and improved vision (best corrected visual acuity). In the long-term assessment, the final visual acuity was better in 11 patients (91.7%) compared to the initial results. On average, 7 days after the surgery, air in the eyeball occupied about one-fifth of the vitreous chamber and was completely absorbed about 10 to 14 days after surgery.

**Conclusions:**

In the above-mentioned study, very good results of treatment of open-rooted retinal detachment were achieved. The air was quickly absorbed and visual acuity improved. Owing to the relatively fast absorption time, this method could be used successfully in high altitude patients and people often travelling by airplane.

## Abstract 2

Supplemental Digital Content is available in the text

## 1 Introduction

Rhegmatogenous retinal detachment is a condition requiring surgical intervention. There are several treatment methods for the above-mentioned disease. Depending on the surface of the detachment, the number and extent of tears, the presence of PVR (proliferative vitreoretinopathy), satisfactory visibility in the fundus of the eye, and an extra (scleral buckling) or intraocular (pars plana vitrectomy [PPV]) technique may be used.

During the vitrectomy, silicone oil and various expansion gases (SF_6_—sulphur hexafluoride, C_2_F_6_—hexafluoroethane, C_3_F_8_—octafluoropropane) can be used for endotamponade.^[1,2]^ Depending on the type of gas used, the absorption time varies.^[3]^ Silicone oil needs to be removed after three months following its administration.

After the surgery with expansion gases, the patient cannot travel by plane or stay at high altitudes because of the high intraocular pressure (IOP) variation. High values of this pressure can lead to eyeball pain, gradual damage to the optic nerve fibres, and consequently to decreased visual acuity.^[4]^ If the patient cannot comply with the conditions then the alternative is a vitrectomy with silicone oil. However, this method also carries the risk of complications such as cataract, secondary glaucoma, and keratopathy.^[5]^

An effective method of treatment of retinal detachment as well as an alternative to the methods mentioned above is the PPV with filtered air.^[6]^

The air remains in the vitreous chamber for a shorter time than the expansion gases, but it seems that it is a sufficient time to achieve an effective retinal reattachment. The aim of this article is to evaluate the effectiveness of treating rhegmatogenous retinal detachment by PPV using filtered air. The article presents the results of the research in a small (12 people), selected group of patients. In the future, it is planned to extend the survey by including a larger number of people and creating a control group.

## 2 Materials and methods

Patients with recent rhegmatogenous retinal detachment were qualified for PPV treatment with filtered air by a vitreoretinal surgeon, who then carried out these procedures. No changes were made after the study began. The exclusion criteria were as follows: eyes after previous vitreoretinal procedures, with other diseases such as proliferative diabetic retinopathy, advanced optic neuropathy n. II, a full-thickness macular hole, an active choroidal neovascularization in the treatment phase, geographic atrophy, aphakia, high degenerative myopia (defect >–10.0 Dsph), with a large tear, with PVR type C1, patients with locomotor system diseases that prevent the appropriate required postoperative position. It is very likely that in eyes with C1 type of PVR it is possible to successfully apply PPV using filtered air. However, a decision was made to subject them to PPV using 25% SF_6_. Patients with fresh retinal detachment of the retina in the upper quadrants were included in the study. For retinal detachments in the lower quadrants, a better method is to use PPV with gas (the gas expands and stays in the eye longer than the air) or oil. In these patients, a lying position with the head placed higher is recommended. The position on the back is recommended in case of using silicone oil, whereas the position on the stomach is applied in case of using gas.

According to the principles of the Helsinki declaration, each patient was informed before the surgery about how the procedure would be performed, about possible complications, and signed the informed consent for the procedure. The study was approved by the Bioethical Commission at the Silesian Medical Chamber, Medical University of Silesia in Katowice (Bioethics Committee approval no. 20/2017).

Each patient also had an ocular ultrasound examination, an IOP measurement with applanation tonometry, and a fundoscopy. The location of the retinal tear, the area occupied by the retinal detachment, and whether the macula was affected or not were determined.

Each procedure was performed under retrobulbar anesthesia by one vitreoretinal surgeon on D.O.R.C Associate. Two types of drugs were administered to each operated eye before the treatment—levofloxacin and tropicamide. Each patient received also dexamethasone after the procedure. Tropicamide was recommended to the patients for 7 days, whereas dexamethasone and levofloxacin for 14 days after the procedure. Depending on the local condition, in some cases, the drugs were used for a longer period of time. During the vitrectomy, 3 ports in the sclera were created. The vitreous body was completely excised with the vitrectomy 20G (the central and peripheral part) to eliminate vitreoretinal tractions which could arise during convalescence. The retina around the openings and peripheral retina were protected by endophotocoagulation. The endofococulation was used to prevent recurrent retinal detachment. At the end of the procedure, filtered air was used as endotamponade in each patient. After removal of the cannula from the ports, the 8.0 sutures were put on the sclera and then it was checked for air leakage. After the procedure, patients were advised to walk with their head down and to lie on their stomach to achieve better effects of the filtered air on the retina.

Each patient was monitored 1, 7, 14, 30, 90, and 180 days after the surgery. The time of air absorption, visual acuity, and IOP were checked and eye fundus examination was performed.

To maintain the patients’ anonymity, each patient was marked with an ordinal number from 1 to 12, including the information about sex (K-female, M-male), age counted in years and a mark for the eye affected by the detachment (Appendix 1). An electronic database in which patients were marked with numbers, without giving any personal data, was created. The results were developed on the basis of the information collected in the database. The patients’ medical records were used under the written consent of the clinic director. The entire original medical documentation is stored at the University Clinical Center in Katowice.

## 3 Results

Twelve eyes (12 patients: 3 women and 9 men) were included in the study. In 9 cases, it was the right eye and in 3 the left eye. These were patients between the ages of 45 and 70 years (average age 59.2). Two (16.7%) patients who had surgery had pseudophakic eyes (number 8 and 10); 4 patients (33.3%) had their own translucent lenses (number 3–5 and number 9); the other 6 patients (50%) had cataracts. In 3 of 6 patients with cataract, during the vitrectomy patients’ own lenses were also removed by phacoemulsification and artificial lenses were implanted. In total, during the surgery, their own lenses were left in 7 cases. Compared to PPV patients who had their own lenses saved, better long-term BVCA results were observed in patients who had a cataract removed during PPV and in pseudophakic eyes (condition before the surgery). Despite the complexity of the procedure, simultaneous cataract removal and PPV did not lead to additional problems or postoperative complications.

Six of 12 eyes were short-sighted. The greatest myopia was –5.5 Dsph, whereas the lowest was –0.25 Dsph. The average time in which the patient noticed the deterioration of vision before the procedure was about 8.3 days (maximum 42 days, minimum 1 day). The more time which had passed since the patient noticed symptoms before surgery, the worse the prognosis after the procedure, especially in patients with retinal detachment with macular involvement.

In the group of patients, retinal detachment covered from 1 to 3 quadrants. In 5 patients, the detachment covered 1 quadrant and in all 5 cases it was the upper temporal quadrant; in another 5 patients 2 quadrants were covered (2 cases—detachment in the upper quadrant, 1 in the nasal quadrant, 1 in the temporal quadrant), whereas in the 2 remaining patients, detachment was observed in 3 quadrants and in both cases they were temporal and upper quadrants. In the majority of eyes, the most common site of retinal detachment was the upper quadrant. Retinal detachment was most often observed in the upper quadrants—upper temporal quadrant (91.7%)—11 cases of retinal detachment. In only 1 case (No. 8), the tear was found in a quadrant other than the upper temporal—lower nasal quadrant (8.3%).

Of all 12 eyes, in 5 (41.7%, No. 1, 4, 6, 7, 11) retinal detachment affected the macula, in the remaining 7 (58.3%) the macula remained unaffected.

Patients were monitored for air absorption and gradual improvement in vision. On average, 1 day after the surgery the vitreous chamber was filled by 90% air, and 7 days after the surgery, the air constituted only about 20%. As early as 7 days after the procedure, 10 patients could freely read letters on the Snellen chart from a distance of 5 m. The degree of absorption of the filtered air used during PPV was examined in the ophthalmoscope. Only 14 days after surgery, the air was completely absorbed in all patients. Patients with the macula not affected by the eye detachment had better outcomes than patients whose macula was affected by the detachment. In all PPV patients, regardless of whether the retinal detachment was macular or non-macular, an improvement in visual acuity was observed.

In the group of patients tested, in 2 cases (No. 5 and 6), there was a periodic increase in IOP. In 1 patient, increased IOP was measured postoperatively on the 7^th^ day (IOP = 32 mmHg) and in the other patient on the 14^th^ day (IOP = 36 mmHg). In these patients, local antiglaucoma medications were started, resulting in a decrease and normalization of IOP. These patients required the use of drops, which lowered IOP for several weeks. After this time, the pressure was normalized and the medication was discontinued. After 6 months, the pressure in the tested group ranged from 11 to 21 mmHg. In 5 patients who underwent vitrectomy with their own lenses saved, a nuclear cataract developed. In 1 patient, the cataract appeared in 1 month after the surgery, in another patient after about 2 months, in 2 patients after about 3 months, and in the last patient after half a year from the retinal detachment surgery. These patients had also cataract surgery within a few months. It follows from the above that although the air used for endotamponade stays in the eye for a short period of time, it does not prevent the development of a cataract. Therefore, it can be concluded that it would be better to use this method of treatment in patients with pseudophakic eyes.

In 1 of 12 patients, 3 months after vitrectomy, an epiretinal membrane appeared in the operated eye (no. 3). In 1 patient 2.5 months after surgery, recurrent retinal detachment (no. 12) in the upper quadrants (with macular involvement) was diagnosed.

In each patient before and after the procedure, an eye ultrasound examination was performed in the B projection. In this way, the detachment before PPV and the retinal application after PPV was confirmed in an indirect study, and the recurrent retinal detachment was depicted. Initially, in 100% of the operated patients there was an anatomic success in the form of retinal reattachment. In 1 patient (no. 10) after 2.5 months, the retina detached again. This patient underwent another vitrectomy with silicone oil.

In a long-term assessment, in 91.7% of operated eyes, BVCA improvement was achieved compared to preoperative BVCA (Table 1).

**Table 1:**
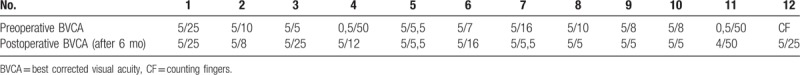
BVCA before and after vitrectomy.

## 4 Discussion

Gases are commonly used for endotamponade during posterior vitrectomy, both in macular holes and epiretinal membrane, as well as in retinal detachment operations. The most commonly used gas is 25% SF_6_. Less often the air is used as an endotamponade. However, it is also a method widely recognized as effective.^[4]^

In a study conducted on a group of 12 people, the effectiveness of the retinal reattachment in the first month was 100%, whereas in the final assessment—after 6 months—the effectiveness was noted in 91.7%. In each patient, retinal laser therapy was performed around the openings and tears as well as on the periphery. The group included only 12 patients, but even with such a small group of patients when evaluating therapeutic effects, it can be seen that PPV with air is a method that brings favorable results in the treatment of rhegmatogenous retinal detachment.

The study provided adequate adhesion of the retina to RPE (retinal pigment epithelium) only within 24 hours after the surgery. Other authors also mention similar results.^[7–9]^ The time at which the air remains in the eyeball after it has been filled in is sufficient to allow for an effective retinal reattachment.

In the study presented, the time for which the air remained in the eyeball did not exceed 14 days. However, even when the air remained in the eye for such a short period of time, this resulted in faster clouding of the lens. Five of the 12 patients developed a nuclear cataract after the procedure and the lens was more-or-less opaque.

PPV with air seems to be a favorable method for rhegmatogenous retinal detachment in a selected group of patients. The air remains for a shorter time in the eye than other gases commonly used in vitrectomy, it is absorbed more quickly from the eyeball and thus patients are more likely to start moving freely in their surroundings, and they can recognize large optotypes on the Snellen chart in a significantly shorter time. Favorable effects are also obtained in patients who live at high altitudes—they can return to their homes earlier—as well as in patients with difficulties in maintaining the suggested position of the body after surgery (patients with the bone-joint problems).^[10–12]^

In the 12-patient study, the results appear to be satisfactory and at the same time encourage more frequent use of air as endotamponade during rhegmatogenous retinal detachment during posterior vitrectomy.

## Author contributions

**Writing – original draft:** Katarzyna Michalska-Małecka, Justyna Sierocka-Stępień, Aneta Michalik-Jakubek, Katarzyna Witek, Katarzyna Nowomiejska, Anselm Jünemann, Robert Rejdak.

**Writing – review & editing:** Katarzyna Michalska-Małecka, Justyna Sierocka-Stępień, Aneta Michalik-Jakubek, Katarzyna Witek, Katarzyna Nowomiejska, Anselm Jünemann, Robert Rejdak.

## Supplementary Material

Supplemental Digital Content
