# Supplementary material for: Evaluation of the treatment results in patients with rhegmatogenous retinal detachment treated by pars plana vitrectomy with air: Preliminary results
Source: Medicine (Baltimore). 2018 Jun 1;97(22):e10902. doi: 10.1097/MD.0000000000010902 (PMC6392625; doi:10.1097/MD.0000000000010902)
Supplement: Supplemental Digital Content [file medi-97-e10902-s001.doc]

Appendix 1. Patients' data: ordinal number, age, sex and eye

1.F, 49 years old, RE

2. M, 59 years old, RE

3. M, 53 years old, RE

4. M, 50 years old, RE

5. M, 65 years old, RE

6. M, 66 years old, LE

7. M, 45 years old, RE

8. M, 57 years old, LE

9. F, 63 years old, RE

10. M, 70 years old, RE

11. M, 63 years old, RE

12. F, 70 years old, LE

F – female, M- male

RE – right eye, LE - left eye
